# Women’s Health: Endometriosis and PCB Exposure

**Published:** 2006-07

**Authors:** Carol Potera

Endometriosis may be related to exposure to persistent pollutants such as polychlorinated biphenyls (PCBs), according to research published in the May 2006 issue of *Chemosphere*. This gynecological disorder linked to infertility afflicts 10% of U.S. women of reproductive age. Researchers measured blood PCB levels in women undergoing laparoscopy for suspected endometriosis or other gynecological conditions. Higher levels of PCBs were detected in women with histologically confirmed endometriosis compared with controls.

Toxicologist Elena De Felip of the Istituto Superiore di Sanità in Rome and her colleagues measured 11 PCB congeners that are most abundant in human tissue. In 80 women aged 20 to 40, the sum of all congeners was 1.6 times higher in the 40 women diagnosed with endometriosis than in controls. Three congeners, PCBs 138, 153, and 180, were particularly higher in women with endometriosis. These three congeners have been reported to have estrogenic activity and to interfere with hormone-regulated processes.

PCBs have been used since the 1930s, mainly in electrical equipment. Although no longer manufactured, these persistent chemicals accumulate in the food chain; today meat, fish, eggs, and milk are chief sources of PCBs. But diet seems unable to explain the difference in PCB levels detected in the two groups of women, since “the dietary habits of the women were basically the same,” says De Felip.

De Felip suspects that differences in how women detoxify and eliminate PCBs from the body may explain the disparity. These processes are mediated by polymorphic enzymes; therefore, she says, differences in toxicokinetic activity may represent the basis for the higher concentrations detected in women with endometriosis and may also be related to higher or lower susceptibility to that condition.

Studies of PCBs and endometriosis face several limitations. Researchers typically measure only a few widespread congeners that are selected because of their toxicological activities, including an association with cancer shown in animal models. “So we’re only getting part of the picture,” says Germaine Buck Louis, chief of the Epidemiology Branch at the National Institute of Child Health and Human Development.

In a study described in the January 2005 issue of *Human Reproduction*, Buck’s team measured 62 congeners in 84 women undergoing laparoscopy. Levels of 4 antiestrogenic congeners were 3.77 times higher in women diagnosed with endometriosis than in controls. “We don’t fully understand the role of estrogenic and antiestrogenic PCBs,” she says, but complex interactions of many PCBs as well as other chemicals may be involved in developing endometriosis.

Recent advances in PCB detection methods allow more congeners to be measured at lower concentrations. “Women with endometriosis may have low levels of a particular congener not found in [other women],” says Louis. Moreover, breastfeeding reduces PCB levels in women, so women without endometriosis may have lower blood levels because they become pregnant and breastfeed more often. “There’s no ideal comparison group for endometriosis studies,” says Louis, and “there are no easy answers.”

